# Cell State Transitions Drive the Evolution of Disease Progression in B-Lymphoblastic Leukemia

**DOI:** 10.1158/2767-9764.CRC-25-0277

**Published:** 2026-01-07

**Authors:** Curtis Gravenmier, Sadegh Marzban, Yi-Han Tang, Nancy Gillis, Bijal D. Shah, Lynn C. Moscinski, Ling Zhang, Jeffrey West

**Affiliations:** 1 Hematopathology and Laboratory Medicine, H. Lee Moffitt Cancer Center & Research Institute, Tampa, Florida.; 2 Integrated Mathematical Oncology, H. Lee Moffitt Cancer Center & Research Institute, Tampa, Florida.; 3 Department of Cancer Epidemiology, H. Lee Moffitt Cancer Center & Research Institute, Tampa, Florida.; 4 Department of Malignant Hematology, H. Lee Moffitt Cancer Center & Research Institute, Tampa, Florida.

## Abstract

Cancer stem cells (CSC) are hypothesized to promote tumor progression through innate chemoresistance and self-renewal. CSCs reside in the CD34^+^/CD38^−^ immunophenotypic subpopulation of acute myeloid leukemia (AML). Isolation of CSCs from B-lymphoblastic leukemia (B-ALL) has proven difficult, and the cells of interest apparently are not isolated to the CD34^+^/CD38^−^ compartment. This may be explained, in part, by temporal variations of CD34 and CD38 expression which result in stochastic cell state transitions (e.g., from CD34^+^/CD38^+^ to CD34^+^/CD38^−^). We present a mathematical model of these transitions and correlate salient findings with *BCR::ABL1* status, minimal residual disease (MRD), and relapse in adult B-ALL. As the CSC hypothesis is well supported in AML, we focus on transitions to and from the hematopoietic stem cell compartment (CD34^+^/CD38^−^). Our analysis suggests the presence of dedifferentiating transitions to a CD34^+^/CD38^−^ stem cell–like immunophenotype, especially in B-ALL with *BCR::ABL1*. In contrast, *BCR::ABL1*-negative patient samples have low CD34^+^/CD38^−^ self-renewal rates and either high CD34^+^/CD38^+^ or CD34^−^/CD38^+^ incoming rates. High CD34^+^/CD38^−^ self-renewal is also associated with positive MRD following induction chemotherapy. We find a lack of observable changes in cell state transitions between diagnosis and relapse specimens. Furthermore, simulated therapies targeting the stem cell–like compartment indicate that blocking transitions to the CD34^+^/CD38^−^ state (i.e., blocking dedifferentiation) is more effective than promoting transitions from the CD34^+^/CD38^−^ state toward other states (i.e., promoting differentiation) to reduce the proportion of CD34^+^/CD38^−^ cells. The modeling framework used here is a novel, useful tool to infer prognosis and genotype from routine flow cytometry.

**Significance:**

Flow cytometry characterization of B-ALL samples (diagnosis, remission, and relapse) is used to parameterize a mathematical model of cell state transition rates and stratify patients for post-induction chemotherapy MRD.

## Introduction

Cancer stem cells (CSC) are hypothesized to drive tumor progression by serving as a drug-resistant reservoir with the capacity for self-renewal. The first empirical evidence to support CSCs was obtained from human acute myeloid leukemia (AML) when it was discovered that the ability to transplant AML to severe combined immune-deficient mice is restricted to leukemia cells with a CD34^+^/CD38^−^ immunophenotype similar to normal hematopoietic stem cells (1). The progeny of transplanted CD34^+^/CD38^−^ leukemia cells acquired lineage-specific markers and reproduced the French–American–British classification of the original human AML specimen, consistent with *in vivo* differentiation. Moreover, serial transplantation of CD34^+^/CD38^−^ leukemia cells demonstrated a capacity for self-renewal, offering strong support that the CD34^+^/CD38^−^ subpopulation encompasses leukemia cells with stem cell–like properties (2). Putative AML CSCs also possess characteristics associated with multidrug resistance. For instance, there is higher ATP-binding cassette transporter expression in CD34^+^/CD38^−^ AML cells compared with CD34^+^/CD38^+^ AML cells (3), and a subset of CD34^+^/CD38^−^ cells is able to resist venetoclax by altering what substrates are used to fuel the tricarboxylic acid cycle and oxidative phosphorylation (4). Accordingly, a high frequency of CD34^+^/CD38^−^ cells at AML diagnosis has a strong negative impact on survival (5–7) and the proportion of AML cells expressing stem cell markers increases between diagnosis and relapse (8).

Isolation of CSCs from B-lymphoblastic leukemia (B-ALL) has proven difficult, perhaps because leukemia-initiating cells are not isolated to the CD34^+^/CD38^−^ compartment (9–12). This may be due to temporal variation of CD34 and CD38 expression which results in stochastic cell state transitions (e.g., from CD34^+^/CD38^+^ to CD34^+^/CD38^−^). Indeed, clonal subcultures derived from single B-ALL cells give rise to subpopulations with disparate CD34 and CD38 expression within hours and ultimately reproduce a heterogeneous leukemia regardless of the initial CD34 and CD38 immunophenotype (13). Monitoring the clonal subcultures using time-lapse epifluorescence microscopy reveals temporal variation of CD34 and CD38 expression by single cells prior to any cell division, confirming that temporal variation results from transient marker expression rather than generation of differentiated progeny (13). These observations conflict with the hierarchical nature of the CSC hypothesis and support that B-ALL subpopulation dynamics are more accurately described by allowing for spontaneous, reversible cell state transitions. Still, a high frequency of CD34^+^/CD38^−^ cells at diagnosis is associated with minimal residual disease (MRD) and poor prognosis in childhood B-ALL (14–16), and a CD34^+^/CD38^−^ immunophenotype is typically observed in B-ALL with *BCR::ABL1*, which constitutes a high-risk subgroup of B-ALL (17).

### Cell state transitions drive the evolution of disease progression

Our study aims to investigate the hypothesis that B-ALL cell state transitions involving CD34 and CD38 carry prognostic significance because these transitions affect the prevalence and stability of the CD34^+^/CD38^−^ cell state. Tools based on single-cell transcriptomics (18, 19) and FACS with flow cytometry (20, 21) have begun to unravel cancer cell state dynamics in experimental systems, but these sophisticated techniques have not yet entered the clinical laboratory. Therefore, we devised an innovative method to infer cell state transition dynamics from clinical flow cytometry data. Transitions between CD34^+^/CD38^−^, CD34^+^/CD38^+^, CD34^−^/CD38^+^, and CD34^−^/CD38^−^ B-ALL cell states were modeled as an irreducible Markov chain. The resulting transition matrix values are useful for predicting important features of B-ALL, including *BCR::ABL1* status and response to induction chemotherapy.

### Mathematical models of cell plasticity

Cancer is a complex, evolutionary disease in which mathematical and computational models are highly appropriate to study and predict disease dynamics (22–24). Previous mathematical models have considered the role of plasticity in treatment-induced stemness (25) and other nongenetic mechanisms of drug resistance (26, 27). Other approaches consider the interplay between Lamarckian, plastic induction of resistance and Darwinian, genetic selection for resistance (28, 29). Mathematical modeling has shown promise in describing the rates of clonal expansion of hematopoietic stem cell variants (30–32) and the dependence on microenvironmental context (33), cytokine signaling (34), aging (35), or hematopoietic cell transplantation (36). Markov chain models are a common mathematical framework to simulate time dynamics of systems with a finite number of states (e.g., cell types) in which spontaneous switching between states occurs at a predictable rate or likelihood (e.g., cell state plasticity; refs. 37–40). For example, cell states may be inferred by lineage information and rates of cell state switching estimated by fitting Markovian models to temporal measurements (41). Similar models extend finite states to allow for a continuous range of possible plastic phenotypes (42, 43) or continuous time (44). The temporal kinetics of AML disease dynamics have been modeled using time-sequential bulk RNA sequencing data to parameterize a state–transition model (45). A similar model uses transcriptomics data to parameterize a state–transition model that predicts response to tyrosine kinase inhibitors in chronic myeloid leukemia (46). Herein, we attempt to estimate cell state transitions involving CD34 and CD38 expression using a novel dataset of patients with B-ALL, along with patients in molecular remission as a control group.

## Materials and Methods

### Patient inclusion criteria

Following institutional review, patients eligible for inclusion in the study were identified by searching the laboratory information system at Moffitt Cancer Center for all instances of a specific 10-color flow cytometry panel performed between May 2016 and May 2023. Each diagnostic flow cytometry result was reviewed to confirm that the relevant panel had been performed on a treatment-naïve specimen and the diagnosis was indeed B-ALL in conjunction with clinical and other pathologic findings. Then, patients selected for inclusion in the study underwent chart review to obtain information, including age, sex, date of diagnosis, molecular genetic and cytogenetic findings, treatment regimen, MRD test results, date of last contact, and date of relapse and/or death, if applicable. This study was conducted under institutional review board (IRB) protocol MCC #22116, which was reviewed and approved by an IRB in accordance with the principles outlined in the Belmont Report. The IRB evaluated the study under the Department of Health and Human Services regulations at 45 CFR 46.104(d)(4) and determined that the project qualifies for exemption from ongoing IRB oversight. Additionally, the IRB granted a full waiver of Health Insurance Portability and Accountability Act (HIPAA) Authorization after confirming that the criteria specified in the HIPAA Privacy Rule [45 CFR 164.512(i)(2)] were met.

Genetic data consisted of a hybrid capture–based next-generation sequencing (NGS) panel (FoundationOne Heme), conventional karyotyping, *BCR/ABL1/ASS1* tricolor FISH, and *MLL* (*KMT2A*) 11q23.3 break-apart FISH as applicable per patient. MRD testing consisted of 10-color flow cytometry, qRT-PCR for *BCR::ABL1* p190 and p210 fusion transcripts, and multiplex PCR and NGS to identify clonal immunoglobulin gene sequences (ClonoSEQ) or gene fusions as applicable per patient. The 10-color flow cytometry panel used for diagnosis and MRD detection was performed on a Gallios System and analyzed using Kaluza software (Beckman Coulter). Antibodies included in the panel are CD15, CD130, CD10, CD58, CD22, CD33, CD13, CD123, CD19, CD20, CD45, CD38, CD34, CD81, and CD200 (BD Biosciences, BioLegend, and Beckman Coulter). A minimum of 750,000 events were collected for MRD assays with a validated lower limit of detection of 0.01%.

### Sample collection and analysis

**Figure 1.**
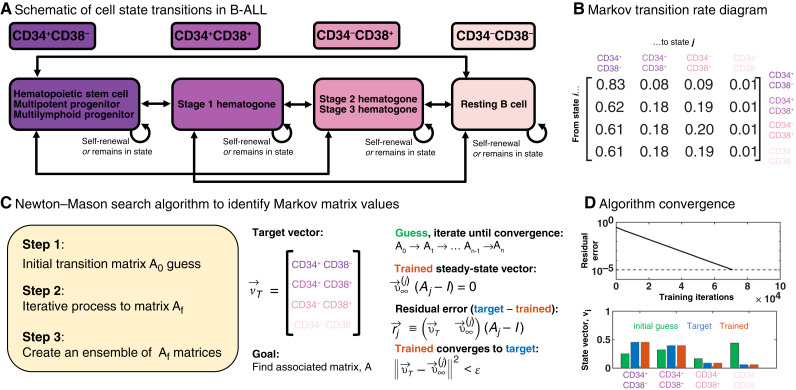
Parameterization process for the Markov chain model. **A,** Schematic of cell states characterized by CD34 and CD38 and the 16 possible transitions between four states. **B,** Markov transition rate matrix which represents the transition probability from the state given by the row into the state given by the column. **C,** Newton–Mason search algorithm identifies Markov matrix parameter values in three steps: initial guess, iteration, and ensemble. **D,** Validation of algorithm convergence. Initial guess has a high residual error, which converges logarithmically toward the target vector until acceptable tolerance is reached (dashed line). Error is measured between steady-state vector v (corresponding to transition matrix M) and the flow cytometry measurements for each patient sample.

The proportion of CD34^+^/CD38^−^, CD34^+^/CD38^+^, CD34^−^/CD38^+^, and CD34^−^/CD38^−^ leukemia cells in diagnostic peripheral blood (PB) and/or bone marrow (BM) specimens was determined using a simple flow cytometry gating strategy that utilized mature neutrophils and lymphocytes as reference populations (Fig. 1). Samples were first analyzed to exclude doublets and debris. Then, mature neutrophils and lymphocytes were identified from a side scatter versus CD45 plot and painted in separate colors. The leukemia population was selected by drawing a polygonal gate to include all CD19-positive, CD45-dim positive to negative events and, if necessary, plasma cells were excluded using an interdependent gate to remove CD38-bright events. Lastly, all cellular events were visualized on a CD34 versus CD38 plot, and the leukemia population was divided into CD34^+^/CD38^−^, CD34^+^/CD38^+^, CD34^−^/CD38^+^, and CD34^−^/CD38^−^ quadrants, with mature neutrophils serving a CD34^−^/CD38^−^ reference population.

### Markov chain model parameterization

We next developed a Markov chain mathematical model to quantify transition rates between the four leukemia cell states defined by CD34 and CD38 expression (Fig. 1). We employed the Newton–Mason iterative, algorithmic search process (47, 48) using flow cytometry characterization of four B-ALL cell states stated above. This numerical search procedure was used to derive patient-specific Markov matrices, describing the stochastic cell state transitions and resulting in estimates for cell state transition rates for each patient. A numerical Markov chain modeling training procedure was performed on flow cytometry data of a cohort of samples of PB (*N* = 46) and BM (*N* = 63) from patients with B-ALL with matched clinical and molecular features such as *BCR::ABL1* status, comprehensive genomic profiling, MRD post-induction chemotherapy, and 3-year relapse. Critical to our goal of quantifying the evolution of state transition rates, we also obtained BM measurements for a cohort of individuals in molecular remission (*N* = 38).

### Markov transition matrix identification

We describe the transition of cells between states using a stochastic transition matrix, *M*, within a Markov dynamic system. The proportion of cells in each of *i* states is contained in the state-vector ${\vec{\upsilon }}_{K}$ for discrete time steps, *K*, such that the state changes according to the following equation:

$${\vec{\upsilon }}_{K+1}={\vec{\upsilon }}_{K}M$$

A well-known property of Markov dynamic systems is that the state-vector at time step *K* can be found directly by

$${\vec{\upsilon }}_{K}=\upsilon_{0}M^{K},$$

in which ${\vec{\upsilon }}_{0}$ is the initial state-vector at time zero. Each matrix has a corresponding steady-state vector defined as

$${\vec{\upsilon }}_{\infty }\left(M-I\right)=\;0,$$

in which *I* is the identity matrix. Here, our state vector contains four states for which index i ∈ [1, 2, 3, 4] corresponds to CD34^+^/CD38^−^, CD34^+^/CD38^+^, CD34^−^/CD38^+^, and CD34^−^/CD38^−^, respectively (see Fig. 1A and B).

### Algorithm to compute Markov transition matrix

Next, we use an iterative search algorithm (Fig. 1C) to determine the most likely Markov matrix, *M*, associated with each patient-specific target vector, ${\vec{\upsilon }}_{T}$. This target vector composition is determined by flow cytometry for each patient at diagnosis before induction chemotherapy. We assume that the target vector represents quasi-steady state disease dynamics, and thus the search algorithm finds a Markov matrix such that the following relation is true:

$${\vec{\upsilon }}_{T}\approx {\vec{\upsilon }}_{\infty }.$$

Following the process outlined by Newton and colleagues (47, 48), there is a three-step process to compute the transition matrix, *M*, which we restate below.

**Step 1**: the initial choice of Markov matrix, *M*. The Newton–Mason training algorithm is nonergodic and thus depends on the initial condition chosen to begin the iterative search. As previously outlined by Newton and colleagues (47, 48), the initial choice should be a rank 2 matrix, in which the row indicating the disseminating phenotype (in our case, the stem-like cell type 1) should match the steady-state distribution of disease samples.

Within each group, the initial Markov matrix is constructed row by row as follows. The entries in the first row match the average flow cytometry values for remission samples (intended to represent the disease-free immunophenotype distribution). The entries in the second, third, and fourth row match the average flow cytometry values for that patient sample subgroup (diagnosis BM, diagnosis PB, remission, and relapse, respectively). Stated formally,

$$M_{1}\;=\;{\vec{\upsilon }}_{\infty ,RM}$$

in which *M*_1_ is the first row of M and ${\vec{\upsilon }}_{\infty ,RM}$ is the steady-state average associated with remission patient samples. For all other rows, *j* ≠ 1 such that

$$M_{j}\;=\;{\vec{\upsilon }}_{\infty ,subgroup}$$

in which ${\vec{\upsilon }}_{\infty ,subgroup}$ is the steady-state average associated with diagnosis BM, diagnosis PB, or remission samples.

The intuition behind this choice is that we begin with a matrix in which stem cells are assumed to transition to other cells at a rate proportional to each cell type in samples from patients with B-ALL. This recapitulates the CSC hypothesis, in which a single stem-like cell state is capable of transitioning toward any other cell state, and nonstem cells transition at rate proportional to each cell type in disease-free molecular remission patients.

**Step 2**: the iteration process to final Markov matrix, *M*_*f*_. Starting with initial Markov matrix *M*_0_, entries are iteratively adjusted by randomized adjustments until the steady-state vector converges to the patient’s target distribution, within some error tolerance. First, the residual error defined at each step *j* of the training process is the difference between the patient’s target vector and the steady-state:

$${\vec{r}}_{j}\;=\;\left({\vec{\upsilon }}_{T}\;-\;{\vec{\upsilon }}_{\infty }\right)\;\left(M_{j}-\;I\right)$$

This residual error vector determines the subsequent adjustments made to the Markov matrix for algorithm step *j+1*. Select a random row of M to modulate. A value of δ is subtracted from the column of M corresponding to the maximum entry of ${\vec{r}}_{j}$, and the same value of δ is added from the column of M corresponding to the minimum entry of ${\vec{r}}_{j}$. The added and subtracted value δ is scaled with the size of the Euclidean norm of the residual vector:

$$\delta ={c\left\|{\vec{r}}_{j}\right\|}^{2},$$

in which *c* is a small constant (here, *c* = 0.0005). The search algorithm iterates *M*_*j*_ until numerical convergence threshold such that ${\left\|{\vec{r}}_{j}\right\|}^{2}<\epsilon \ll 1$, for which typically we set $\epsilon =10^{-5}$ (Fig. 1D).

**Step 3**: creating an ensemble of *M*_*f*_ matrices. The algorithm presented in step 2 is randomized (due to randomized row selection), and thus, the training process is inherently stochastic. As reported previously, because the training algorithm is stochastic, the entries of *M*_*f*_ should be thought of as having an associated probability distribution, with a sample mean and variance obtained by training an ensemble of matrices. We repeat the training process, with identical *M*_0_ many times (typically *N* = 100), and average the Markov entries to obtain the sample means.

### Continuous-time Markov chain simulation

We next derived patient-specific continuous time dynamics from discrete transition matrix M estimated from longitudinal flow cytometry. A continuous time generator was obtained as

$$Q\;=\;\frac{1}{∆t}\;\log\left(M\right)\;\;\;\;\left(∆t=1\right)$$

enforcing generator validity by clipping small negative off-diagonals and adjusting diagonals so each column summed to zero:

$$Q_{ij}\leftarrow \;\max\;\left(Q_{ij},0\right)\;\;\;\;\left(i\;\neq j\right),\;\;\;\;Q_{ij}=\;-\underset{i\neq j}{\sum }Q_{ij}$$

The continuous-time Markov chain (CTMC) master equation becomes

$$\dot{\vec{p}}\;=\;Q\vec{p}$$

in which ${\vec{p}}_{i}\;\left(t\right)$ is of length 4 with index i ∈ [1, 2, 3, 4] corresponding to CD34^+^/CD38^−^, CD34^+^/CD38^+^, CD34^−^/CD38^+^, and CD34^−^/CD38^−^, respectively.

### Dimensionality reduction

To reduce dimensionality, states 2 (CD34^+^/CD38^+^) and 4 (CD34^−^/CD38^−^) were aggregated into a single (other) compartment, O, yielding a three-state CTMC with transitions between stem like (CD34^+^/CD38^−^), differentiated (CD34^−^/CD38^+^), and O. We define

$$q\left(O\;\to \;1\right)\;=\;q_{1O}=\;\omega_{2}\;q_{12}\;+\;\omega_{4}\;q_{14},\;\;\;\;q\left(O\;\to \;3\right)\;=\;q_{3O}=\;\omega_{2}\;q_{32}\;+\;\omega_{4}\;q_{34}.$$

in which *ω*_2_ and *ω*_4_ are defined using the stationary weights of two versus four to preserve the steady-state distribution:

$$M^{T}\;\pi =\;\pi ,\;\;\;\;\pi_{i}\;\geq 0,\;\;\;\;\overset{4}{\underset{i=1}{\sum }}\pi_{i}=1$$

$$\omega_{2}=\;\frac{\pi_{2}}{\pi_{2}+\;\pi_{4}},\;\;\;\;\omega_{4}=\;\frac{\pi_{4}}{\pi_{2}+\;\pi_{4}},\;\;\;\omega_{2}+\;\omega_{4}=1.\;$$

Outflow to O from state k (1 or 3) is the sum of parallel exits to 2 and 4:

$$q\left(1\;\to \;O\right)\;=\;q_{O1}=\;q_{21}\;+\;q_{41},\;\;\;\;q\left(3\;\to \;O\right)\;=\;q_{O3}=\;q_{23}\;+\;q_{43}.$$

Direct transitions between 1 and 3 are unchanged by aggregation:

$$q\left(1\;\to \;3\right)\;=\;q_{31},\;\;\;\;q\left(3\;\to \;1\right)\;=\;q_{13}$$

Let p_1_(t) and p_3_(t) denote the fractions in states 1 and 3 with

$$p_{O}\;\left(t\right)\;=\;1\;-\;p_{1}\;\left(t\right)\;-p_{3}\;\left(t\right)$$

We calculated the rates between these states and the population three-state dynamics satisfy

$${\dot{p}}_{1}\;=\;q_{10}p_{0}\;-\;\left(q_{01}\;+\;q_{31}\right)\;p_{1}\;+\;q_{13}p_{3}$$

$${\dot{p}}_{3}\;=\;q_{30}p_{0}\;-\;\left(q_{03}\;+\;q_{13}\right)\;p_{3}\;+\;q_{31}p_{1}$$

For simulations, we integrated the ordinary differential equations (MATLAB ode45) to steady state per patient using their inferred rates. To model reduced self-renewal of the stem-like pool without introducing birth within state 1, we implemented differentiation-promoting interventions by increasing the outflow rates from state 1 (q_13_ and q_1O_), as well as blocking dedifferentiation by reducing inflow rates to state 1 (q_31_ and q_O1_).

## Results

**Figure 2.**
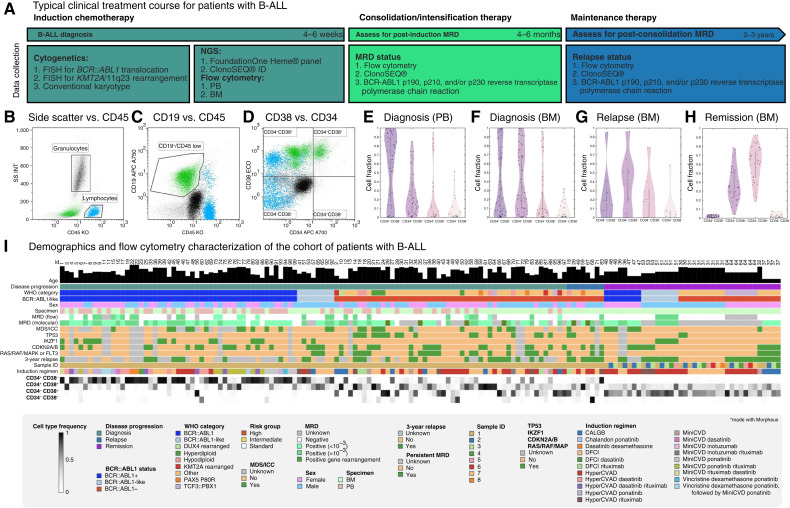
Data collection for B-ALL. **A,** Diagnostic flow cytometry, NGS, and cytogenetic data were obtained for each patient prior to beginning induction chemotherapy and included the elements listed above. MRD test results were also obtained throughout each patient’s clinical course, and instances of morphologic relapse and death were documented to determine the potential impact of B-ALL subpopulation dynamics on therapy response and clinical outcomes. **B–D,** A simple flow cytometry gating strategy was used to select the leukemia population and divide it into CD34^+^/CD38^−^ (hematopoietic stem cell–like), CD34^+^/CD38^+^ (hematogone-like, stage 1), CD34^−^/CD38^+^ (hematogone-like, stages 2 and 3), and CD34^−^/CD38^−^ (naïve B cell–like) subpopulations. Neutrophils served as a reference CD34^−^/CD38^−^ population. **E–H,** The distribution of these subpopulations across the dataset is presented using violin plots. **I,** B-ALL Demographics and flow cytometry characterization of the cohort of patients with B-ALL at three stages in disease progression: diagnosis, relapse, and remission. Demographic information (sex and age) is shown for patients with matched clinical outcomes (MRD status and 3-year relapse), select genetic mutations (*BCR::ABL1*, *TP53*, *IKZF1*, *CDKN2A/B*, RAS/RAF/MAPK pathway, or *FLT3*), World Health Organization (WHO) category, sample type, and flow cytometry characterization. MRD is defined using multiple modalities including flow cytometry and molecular methods. DFCI, Dana-Farber Cancer Institute Consortium protocol for induction chemotherapy; ICC, International Consensus Classification; MDS, myelodysplastic syndrome.

A general outline of the clinical and laboratory management of adult B-ALL is provided in Fig. 2A. Treatment begins with induction chemotherapy, after which the patient is assessed for MRD using sensitive assays, including specialized flow cytometry and clonal immunoglobulin gene sequences (ClonoSEQ). PB and BM specimens can be assessed for flow cytometric and molecular genetic evidence of residual/recurrent B-ALL throughout a patient’s treatment course in order to guide therapy decisions.

The flow cytometry gating strategy that was applied to leukemia cells is shown in Fig. 2B–D. Mature neutrophils and lymphocytes, identified from side scatter versus CD45, served as reference populations to gauge antigen expression. The leukemia population was selected using a polygonal gate to include all CD19-positive, CD45-dim positive to CD45-negative cellular events. Then, the population of interest was divided into quadrants based on CD34 and CD38 expression, with neutrophils acting as a reference CD34^−^/CD38^−^ population. In this way, each B-ALL was described as a composite of four cell states as follows: CD34^+^/CD38^−^ (hematopoietic stem cell–like), CD34^+^/CD38^+^ (hematogone-like, stage 1), CD34^−^/CD38^+^ (hematogone-like, stages 2 and 3), and CD34^−^/CD38^−^ (naïve B cell–like). Figure 2E–H shows these subpopulations in samples from BM (*N* = 63) and PB (*N* = 46) of patients with B-ALL, as well as BM samples from a cohort of individuals in molecular remission for comparison.

The dataset is summarized in Table 1 and visualized in Fig. 2I, in which each row contains patient clinical information (e.g., age, genomic markers, and specimen type), along with flow cytometry characterization (CD34 and CD38 status). Each column is an individual patient, grouped from left to right into samples taken at diagnosis, relapse, or remission. The median age of the cohort was 55 years (IQR, 41–70). There was a balanced mix of *BCR::ABL1*-positive versus -negative samples and PB versus BM samples. All patients had a sample collected at diagnosis, and 32 patients (34%) had a sample from one or two additional clinical time points. Samples from patients in remission provided a comparator group for the leukemic patients at diagnosis or relapse. At diagnosis, *BCR::ABL1*-positive and *BCR::ABL1*-like patients had a higher proportion of CD34^+^CD38^−^ cells, whereas *BCR::ABL1*-negative patients typically had a lower proportion of CD34^+^/CD38^−^ cells and higher CD34^+^/CD38^+^ or CD34^−^/CD38^+^ cell fraction. Patients in remission displayed a high frequency of CD34^−^/CD38^+^ cells.

**Table 1.** Description of the cohort of patients with B-ALL.

Patient characteristic	*N* = 95^a^
Age	55 (41, 70)
WHO classification	​
*BCR::ABL1*	44 (46%)
*BCR::ABL1*-like	6 (6.3%)
Other	31 (32%)
*TCF3::PBX1*	2 (2.1%)
Hypodiploid	3 (3.2%)
Hyperdiploid	3 (3.2%)
*KMT2A* rearranged	3 (3.2%)
*PAX5* P80R	2 (2.1%)
*DUX4* rearranged	1 (1.1%)
Specimen type	​
BM	49 (52%)
PB	32 (34%)
BM + PB	14 (15%)
MRD	​
Yes	68 (72%)
No	26 (28%)
Unknown	1
Persistent MRD^b^	​
Yes	13 (36%)
No	23 (64%)
Unknown	32
Mutation^c^	​
*IKZF1*	14 (16%)
*CDKN2A/B*	31 (36%)
RAS/RAF/MAPK pathway or *FLT3*	19 (22%)
*TP53*	14 (16%)
Unknown	10
Collection time points	​
Diagnosis only	63 (66%)
Diagnosis + relapse	22 (23%)
Diagnosis + remission	4 (4.2%)
Diagnosis + remission + relapse	6 (6.3%)

Abbreviation: WHO, World Health Organization.

a Median (IQR); *n* (%).

b % calculated based on the number of patients with persistent MRD prior to relapse or for at least 3 years of clinical follow up (*n* = 68).

c Patients can have mutations in more than one category.

### Patients in molecular remission are associated with low CD34^+^CD38^−^ cell self-renewal

We employ a Markov chain–based mathematical modeling approach to estimate cell state transition rates among four distinct cell states, characterized by CD34 and CD38 flow cytometry markers (see “Materials and Methods” for details on model parameterization). The model encompasses 16 possible transitions, accounting for all theoretically possible connections between the four cell states (although many transitions may be negligible). Each transition is denoted as M_ij_, in which i represents the target state and j represents the initial state. For example, M_11_ corresponds to self-renewal within state 1 (the stem cell–like state).

**Figure 3.**
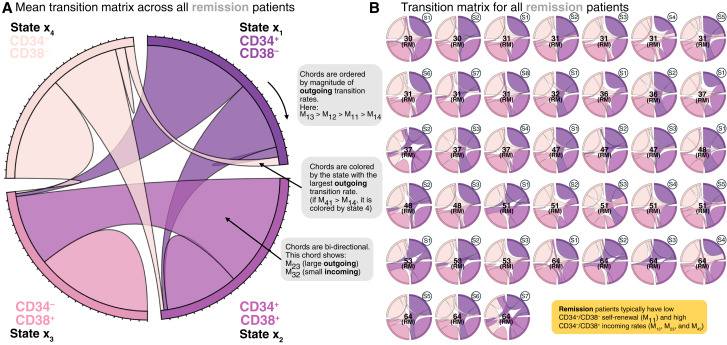
Samples from patients in molecular remission. **A,** Chord diagram illustrating the mean transition matrix across all patients. The chord is organized into four sections (CD34^+^/CD38^−^, CD34^+^/CD38^+^, CD34^−^/CD38^+^, and CD34^−^/CD38^−^), with the size of each chord proportional to the magnitude of the outgoing transition rate. Chords are ordered (clockwise) by the magnitude of outgoing transition by each section for visual interpretation, and each bi-directional chord is colored by which is greater: M_ij_ or M_ji_. **B,** Chord diagram shown for each individual patient sample. Central numbers indicate sample ID, corresponding to the Morpheus diagram in Fig. 2I. Numbers at the top right of each diagram indicate the number of samples available for that patient.

We begin by estimating cell state transition rates for patients in molecular remission, visualized using a chord diagram in Fig. 3A, which shows the mean transition matrix or typical cell state kinetics, averaged across all remission patients.

As the CSC hypothesis is well supported in AML, we begin by making observations on transitions to and from the hematopoietic stem cell compartment (CD34^+^/CD38^−^) in this B-ALL dataset. Patients in remission typically have a low CD34^+^/CD38^−^ renewal rate, M_11_. This low degree of CD34^+^/CD38^−^ cell self-renewal is generally paired with a high degree of transitions into the CD34^−^/CD38^+^ cell state and reflects the normal maturation of hematogones in BM. This provides a baseline for the cell state transitions to compare with patients with leukemia. We also note that there is a moderate degree of interpatient heterogeneity across all patients (Fig. 3B).

### Matched PB and BM samples

**Figure 4.**
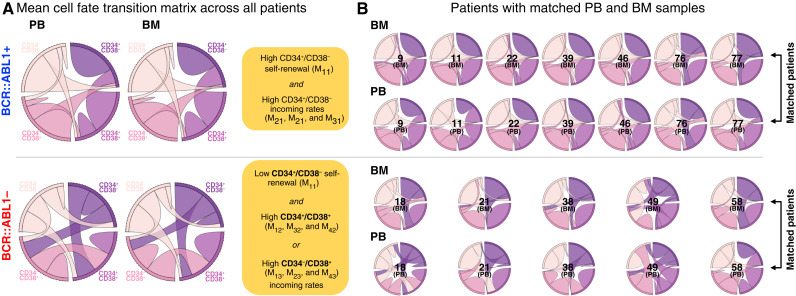
Samples from patients with leukemia. **A,** Chord diagram illustrating the mean transition matrix across all patients, for all PB (left) and BM samples (right), repeated for *BCR::ABL1*-positive (top) and *BCR::ABL1*-negative (bottom) patients. *BCR::ABL1*-positive samples are typically associated with high CD34^+^/CD38^−^ self-renewal (M_11_) and high CD34^+^/CD38^−^ incoming rates (M_21_, M_21_, and M_31_). *BCR::ABL1*-negative patient samples have low CD34^+^/CD38^−^ self-renewal rates (M_11_) and either high CD34^+^/CD38^+^ (M_12_, M_32_, and M_42_) or high CD34^−^/CD38^+^ (M_13_, M_23_, and M_43_) incoming rates. **B,** Matched samples from PB and BM reach good agreement in transition rates for both *BCR::ABL1*-positive or -negative samples. Numbers indicate sample ID, corresponding to the Morpheus diagram in Fig. 2I.

Next, we estimate cell state transitions for samples from patients with B-ALL at diagnosis (Fig. 4). The mean transition matrix (Fig. 4A) for *BCR::ABL1*-positive patients shows high CD34^+^/CD38^−^ cell self-renewal (M_11_) and high CD34^+^/CD38^−^ incoming rates (M_21_, M_21_, and M_31_). In contrast, *BCR::ABL1*-negative patients have low CD34^+^/CD38^−^ cell self-renewal rates (M_11_) and one of the following characteristics: high CD34^+^/CD38^+^ incoming rates (M_12_, M_32_, and M_42_) or high CD34^−^/CD38^+^ incoming rates (M_13_, M_23_, and M_43_). Visually, there is a moderate degree of interpatient heterogeneity across all samples (Fig. 4B).

A subset of patients has matched PB and BM leukemia samples, enabling direct comparison of specimen types (Fig. 4B). Model-predicted cell state transition kinetics are remarkably similar across PB and BM leukemia samples within the same patient, and thus, we are unable to reject the null hypothesis that PB and BM samples are drawn from an identical distribution for all 16 transition rate parameters (see Supplementary Figs. S1–S3; Supplementary Tables ST1–ST6). Therefore, for the rest of the analyses, we will group PB and BM samples together.

### Stem cell hypothesis revisited

**Figure 5.**
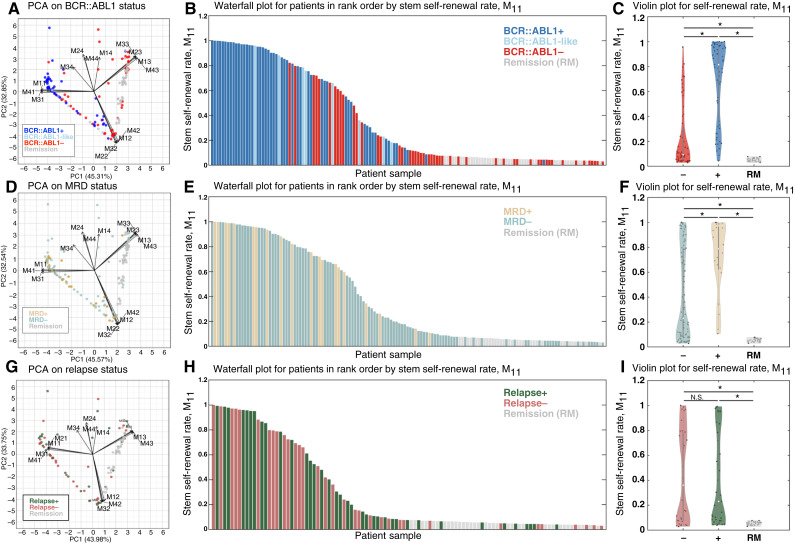
CD34^+^/CD38^−^ self-renewal rate correlates with *BCR::ABL1* status and MRD. **A,** PCA of all Markov transition rate parameters, M_ij_, color-coded by *BCR::ABL1* status. Arrows indicate the loadings for each parameter, representing the correlation between the variables and the principal components. *BCR::ABL1*-positive patients tend to cluster near loadings for transition parameters back into the stem cell state, M_11_, M_21_, M_31_, and M_41_. **B,** Waterfall plot for patients in rank order by the value of stem cell self-renewal transition rate, M_11_. **C,** Violin plot for self-renewal rate, M_11_, categorized by *BCR::ABL1* status, showing statistically different values. **D,** PCA color-coded by MRD outcomes. **E,** Waterfall plot rank ordered by stem self-renewal transition rate, M_11_, color-coded by MRD outcome. **F,** Violin plot for stem self-renewal transition rate, M_11_, color-coded by MRD outcome. **G,** PCA color-coded by relapse. **H,** Waterfall plot rank ordered by stem cell self-renewal transition rate, M_11_, color-coded by relapse. **I,** Violin plot for stem cell self-renewal transition rate, M_11_, color-coded by relapse. The percentage of variance explained differs between panels because not all patients had MRD or relapse data available, and thus, PCA was performed on slightly different patient subsets. *, Statistical significance using two-sample *t* test with significance value of 0.05.

Next, we observe the relationship between important cell state transition values for each patient and correlate salient findings with patient classifications (*BCR::ABL1* status) and clinical outcomes (MRD and relapse). Beginning with *BCR::ABL1*, we applied principal component analysis (PCA) to the 16 Markov transition parameters to visualize patterns of covariation and to explore whether these features show trends of separation by clinical status (Fig. 5A). We note that the Markov transition parameters are constrained by probability normalization (each row summing to one). Thus, these features are not fully independent. Our use of PCA is exploratory, aimed at identifying dominant axes of variation and correlations among transition parameters rather than establishing formal independence. The PCA projects each patient sample onto two axes (PC1 and PC2), which are linear combinations of the 16 input features. The arrows represent the contribution of each input parameter (M_ij_) and indicate how strongly each parameter influences the principal components.

We can make several important observations from this PCA. The vectors [Fig. 5A (black arrows)] representing transition parameters cluster tightly with parameters from the same incoming compartment. For example, M_11_ clusters with the CD34^+^/CD38^−^ state’s other incoming parameters (M_21_, M_31_, and M_41_), meaning that if one of these parameters is high (or low), then the others are likely to be similarly high (or low). Although the PCA does not yield perfectly distinct clusters by *BCR::ABL1* status, it reveals a tendency for *BCR::ABL1*-positive patients to group apart from *BCR::ABL1*-negative patients and remission samples.

Because all incoming transition parameters into a given state covary, it is sufficient to focus on the stem-like self-transition rate M_11_ to gain insights into the CD34^+^/CD38^−^ compartment. The waterfall plot shown in Fig. 5B shows a consistent rank-order relationship of *BCR::ABL1*-positive (high M_11_) and *BCR::ABL1*-negative (mid-range M_11_) to remission samples (low M_11_). Strikingly, remission patients have much lower transition rates into the CD34^+^/CD38^−^ and CD34^−^/CD38^−^ cell states than corresponding samples from patients with leukemia not in remission (See Supplementary Fig. S2). The violin plot confirms the statistical significance of the relationship between self-renewal rate and *BCR::ABL1* status, with a baseline of comparison with molecular remission patients also shown (Fig. 5C). Violin plots for all rates are shown in Supplementary Figs. S2A–S2C and S3A–S3D.

Next, we repeat the PCA and waterfall for post-induction chemotherapy MRD status (Fig. 5D–F) and 3-year disease relapse status (Fig. 5G–I). Here, MRD is defined by a threshold frequency of 10^−3^ leukemia cells detected using specialized flow cytometry and relapse is defined as morphologic relapse. As with *BCR::ABL1*, PCA did not yield sharp clustering of patients by outcomes, but it again highlighted the importance of M_11_. Patients with higher self-renewal transition rates were more likely to have positive MRD after induction therapy (Fig. 5F), whereas no significant differences were observed with respect to relapse (Fig. 5I), consistent with previous literature attempting to predict relapse/recurrence in childhood B-ALL (49, 50).

### Matched diagnosis and relapse samples indicate relatively stable cell state kinetics

**Figure 6.**
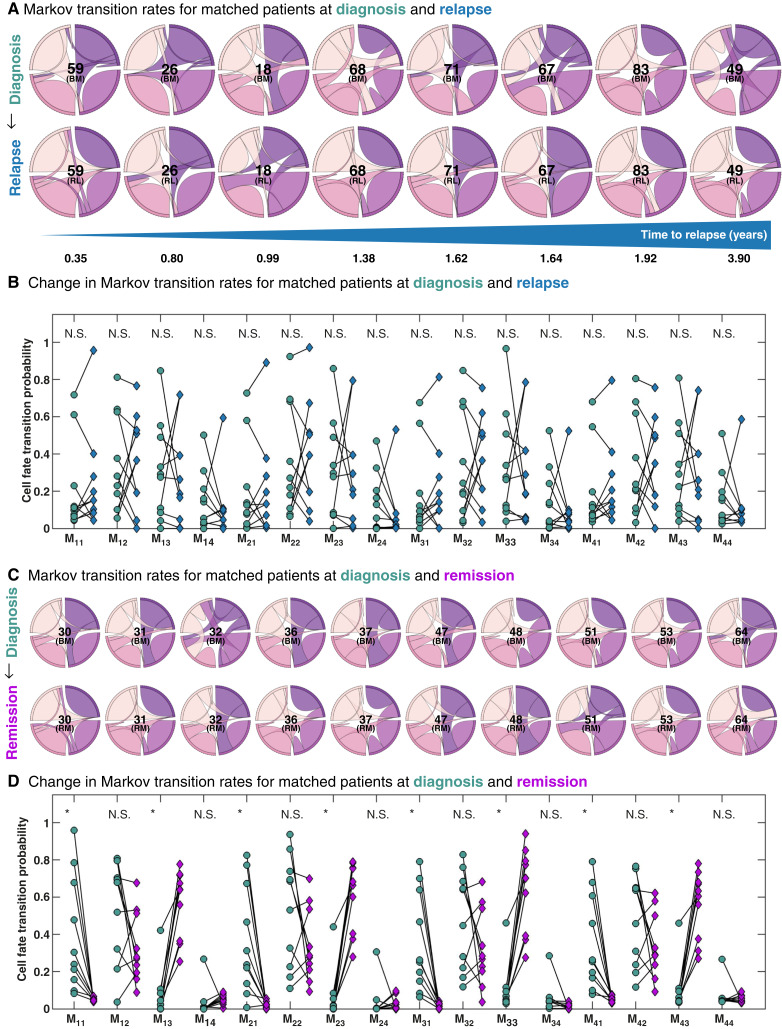
Matched samples from patients at diagnosis and relapse. **A,** Markov transition rate chord diagrams for matched patients at diagnosis (top) and relapse (bottom), ordered from left to right by time to relapse. **B,** Biplot showing the change in each transition parameter value at diagnosis and relapse (left to right). Note that all 16 transition parameters are not significantly different, indicating no clear pattern of change between diagnosis and relapse across our cohort of matched samples. **C,** Markov transition rate chord diagrams for matched patients at diagnosis (top) and remission (bottom). **D,** Biplot showing the change in each transition parameter value at diagnosis and remission (left to right). Significant changes are observed in all transitions back into compartments 1 and 3 (CD34^+^/CD38^−^ and CD34^−^/CD38^+^, respectively). Numbers in **A** and **B** indicate sample ID, corresponding to the Morpheus diagram in Fig. 2I. *, Statistical significance using two-sample *t* test with significance value of 0.05.

We sought to understand whether disease cell state kinetics remain stable before and after treatment for patients who relapse. To address this, we also trained the model using patient samples taken at the point of relapse, which allowed us to directly compare cell state transition rates between diagnosis and relapse time points. Although there is significant variation among patients (Fig. 6A), we do not observe any clear, statistically significant differences between diagnosis and relapse (Fig. 6B). In contrast, matched samples from patients in molecular remission (Fig. 6C) show consistent differences in cell state transition rates (Fig. 6D), particularly in transitions to compartments 1 (CD34^+^/CD38^−^) and 3 (CD34^−^/CD38^+^). Incoming CD34^+^/CD38^−^ transition rates are lower among patients in remission compared with patients at diagnosis, whereas incoming CD34^−^/CD38^+^ transition rates are higher among patients in remission. Although this might simply reflect the presence versus absence of B-ALL, differences in cell state dynamics between diagnosis and remission samples may yield insights into potential mechanisms of differentiation-based therapy, including what transitions make the most effective target.

### Inhibiting dedifferentiation is more effective than promoting differentiation to reduce CD34^+^/CD38^−^ cells

To complement our discrete-time analyses, we implemented a CTMC (see “Materials and Methods”) to quantify patient-specific transition rates between CD34^+^/CD38^−^ (stem cell like; state 1), CD34^−^/CD38^+^ (differentiated; state 3), and other states (O). Each transition rate is denoted as q_ij_, in which i represents the target state and j represents the initial state. For example, q_31_ corresponds to differentiation from state 1 (the stem cell–like state) to state 3 (differentiated cells). This framework allowed us to observe how relapse and remission samples differ in arriving at steady-state proportions of stem cell–like and differentiated cell subpopulations, as well as to test the impact of potential approaches in reducing CD34^+^/CD38^−^ cells.

**Figure 7.**
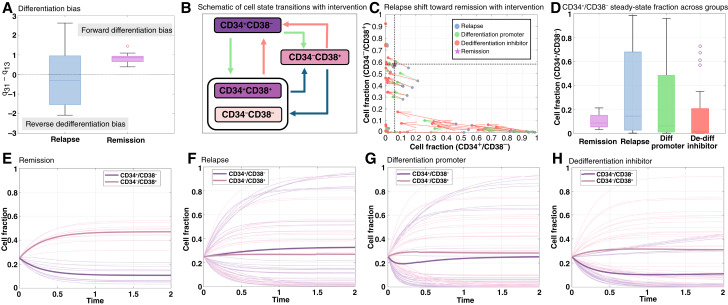
Patient-specific CTMC modeling gives insights about responses to possible intervention. **A,** Remission patients display consistently positive differentiation bias (q_31_ − q_13_ > 0), whereas relapse patients show higher variance and frequent negative values, indicating dedifferentiation. **B,** Schematic of cell state transitions and the proposed classes of intervention. A differentiation-promoting therapy increases transitions from CD34^+^/CD38^−^ cells to the other states (green arrows), whereas a dedifferentiation-inhibiting therapy reduces transitions toward the CD34^+^/CD38^−^ state (red arrows). **C,** For each relapse patient, long-term CTMC states (CD34^−^/CD38^+^ and CD34^+^/CD38^−^) move closer to the remission medians (magenta star, dashed lines) after promoting differentiation or blocking dedifferentiation. **D,** At steady state, relapse patients show increased CD34^−^/CD38^+^ fractions and higher variance compared with remission; simulated interventions partially restore this balance, with dedifferentiation inhibition showing the stronger effect. On the bottom row, time-course simulations of the CTMC model illustrate that (**E**) remission patients converge to a differentiated cell composition with low stem cell–like fraction, (**F**) relapse patients maintain higher stem cell–like fractions, and (**G** and **H**) promoting differentiation and inhibiting dedifferentiation redirect relapse dynamics toward remission-like outcomes. Importantly, inhibiting dedifferentiation transitions is a more effective approach to reducing the stem cell–like compartment.

Across remission patients, the difference between differentiation (q_31_) and dedifferentiation (q_13_) was consistently positive (Fig. 7A), indicating that differentiating transitions dominate over dedifferentiating ones as would be expected in normal BM or PB. Across relapse specimens, however, q_31_ − q_13_ often produced a negative value and displayed markedly higher variance (Fig. 7A). This suggests that CD34^+^/CD38^−^ cells either persist during therapy or are re-established through dedifferentiation of therapy-resistant cells in other compartments and also shows the existence of heterogeneous mechanisms of relapse under therapy pressure. The box plots of all transitions between CD34^+^/CD38^−^ (stem cell like; state 1), CD34^−^/CD38^+^ (differentiated; state 3), and other states (O) for remission and relapse patients are provided in Supplementary Fig. S4A–S4F.

We next asked whether targeting self-renewal of the stem cell–like state by (i) promoting differentiation from the CD34^+^/CD38^−^ state into other states or (ii) blocking dedifferentiation of non-CD34^+^/-CD38^−^ states (see schematic in Fig. 7B) could shift relapse patients toward a remission-like distribution. In the discrete formulation, self-renewal was expressed by M_11_, which is large when outgoing transitions from state 1 are low. Therefore, in the CTMC, we interpret therapeutic reduction of self-renewal as increased outgoing transitions from state 1, which means higher values of q_O1_ and q_31_ (differentiation). Simulations confirmed that both strategies i.e., promoting differentiation and blocking dedifferentiation reduced CD34^+^/CD38^−^ fractions and increased CD34^−^/CD38^+^ fractions, thereby shifting relapse parameterizations toward remission medians (Fig. 7C). Remarkably, blocking dedifferentiation was much more effective than promoting differentiation to shift the steady state toward a remission-like distribution (Fig. 7D). Time-course simulations showed that remission patients converge rapidly to a differentiated-dominated composition (Fig. 7E), whereas relapse patients maintain stem cell–like fractions with slower, more variable dynamics (Fig. 7F). This slow dynamic likely reflects the opposing contributions of forward differentiation (q_31_ and q_O1_) and dedifferentiation (q_13_ and q_1O_). Interventions that promote differentiation are initially effective at reducing CD34^+^/CD38^−^ cells but the population rebounds, limiting the effectiveness (Fig. 7G). Interventions that inhibit dedifferentiation to the CD34^+^/CD38^−^ cell state are not associated with the same rebound and generally achieve a lower CD34^+^/CD38^−^ cell fraction in the steady state (Fig. 7H).

## Discussion

In this work, we employed a Markov chain mathematical model to quantify the rates of cell state transitions between CD34^+^/CD38^−^, CD34^+^/CD38^+^, CD34^−^/CD38^+^, and CD34^−^/CD38^−^ leukemia subpopulations in patients with B-ALL. Such transitions have been observed *in vitro* using time-lapse fluorescence microscopy and cell sorting but have not been captured in the clinical laboratory, primarily because diagnostic flow cytometry and IHC represent a snapshot of the malignant population at a single point in time. Our method can infer subpopulation dynamics for any hematolymphoid malignancy for which clinical flow cytometry data are available. It may also be possible to apply a similar approach wherever quantitative markers are reported in pathology, for instance, hormone receptor expression in breast carcinoma or PD-L1 expression in non–small cell lung carcinoma.

In B-ALL, we find that subpopulation dynamics are useful to predict *BCR::ABL1* status and post-induction MRD (threshold frequency of 10^−3^ leukemia cells detected using specialized flow cytometry). Our analysis suggests the existence of dedifferentiating transitions to a CD34^+^/CD38^−^ stem cell–like immunophenotype in leukemia samples collected from patients with B-ALL, and the tendency for these transitions is especially strong in B-ALL with *BCR::ABL1*. Also of note, transition values were found to be nonzero for all but one patient (see Supplementary Tables ST1–ST3). We interpret these findings as contrary to the hierarchical nature of the CSC hypothesis. Instead, the results favor a nonhierarchical relationship between the leukemia stem cell compartment (CD34^+^/CD38^−^) and other cell states, such that the mathematical modeling predicts that it is possible to access all cell states from any cell state. This has implications for therapies targeting CSCs because CSCs would be replenished over time by transitions from nonstem cells unless the transitions are somehow inhibited.

The results of the study also have practical uses for pathologists and oncologists, such as predicting *BCR::ABL1* when a sample is inadequate for molecular genetic and cytogenetic studies. As *BCR::ABL1*-like patients cluster with *BCR::ABL1*-positive patients in our analysis, it may also be possible to predict *BCR::ABL1*-like B-ALL without testing for specific kinase-activating rearrangements such as *IGH*::*CRLF2*, as long as the *BCR::ABL1* status is known. Genotypic findings of lesser clinical significance also show significant associations with the model results. For instance, it is known that *BCR::ABL1* and *TP53* mutations are almost mutually exclusive in acute lymphoblastic leukemia and, interestingly, patients with these two genetic findings in our study generally have distinct immunophenotypes: a dominant CD34^+^/CD38^−^ subpopulation if *BCR::ABL1* positive versus a combination of CD34^+^/CD38^+^ and CD34^−^/CD38^+^ cells if *TP53* mutant.

Unfortunately, the model parameters were unable to predict relapse after 3 years. We note that this null result is consistent with other findings in literature (49) and suggests that temporal disease evolution occurring during the treatment course may not be reflected in routine clinical flow cytometry data. Our original hypothesis was that cell states (CD34^+^/CD38^−^, CD34^+^/CD38^+^, CD34^−^/CD38^+^, and CD34^−^/CD38^−^) may have different survival rates during therapy, so we should observe treatment-based selection that maximizes transitions to the most therapy-resistant cell state, while minimizing transitions to the least therapy-resistant cell state. There were no statistically significant differences between cell state transitions in paired diagnosis and relapse specimens, but it is unclear whether this is due to the relatively small number of eligible paired specimens or perhaps the subpopulations do not actually differ significantly in terms of fitness. Although leukemia stem cells have been reported to increase in frequency during treatment in AML (8), CD34 and CD38 were not among the markers observed to increase, raising a further possibility that our choice of markers precluded the observation of treatment-based selection. The application of our method to additional markers and types of neoplasia may yet produce clinically significant results. Some limitations of our modeling approach that can be revisited in future works include the assumption that immunophenotypes are in a quasi-steady state, as well as the assumption that transitions are memoryless (an explicit assumption of Markov models). Designating a cell as CD34^+^/CD38^+^, CD34^−^/CD38^−^, CD34^+^/CD38^−^, or CD34^−^/CD38^+^ depends critically on the flow cytometry gating, and changes to the gating strategy will propagate to the Markov chain training algorithm. The Markov model training algorithm is able to recapitulate the flow cytometry measurement within any arbitrary tolerance. Thus, the Markov model training algorithm is only as accurate as the flow cytometry data. This is illustrated in Fig. 2D, which describes the accuracy convergence of the Markov transition model to the flow cytometry data, in which the training process allows the user to specify the desired accuracy between the model’s output (trained vector) and the flow cytometry data (target vector). The accuracy between the Markov steady state and measured flow cytometry data can be arbitrarily low (10^−5^ is used throughout this article).

Lastly, our modeling approach suggests that targeting CD34^+^/CD38^−^ self-renewal represents a promising treatment strategy and that blocking transitions to the CD34^+^/CD38^−^ state (i.e., inhibiting dedifferentiation) is more effective than promoting transitions from the CD34^+^/CD38^−^ state toward other states (i.e., differentiation) to reduce the proportion of CD34^+^/CD38^−^ cells. A simulated therapy that promotes differentiation is initially effective at reducing the proportion of CD34^+^/CD38^−^ cells, but the stem cell–like population rebounds after a short time (Fig. 7G), limiting its effectiveness. This rebound is not observed for a simulated therapy that blocks dedifferentiating transitions, and so it is able to achieve a lower proportion of CD34^+^/CD38^−^ cells in the steady state (Fig. 7H).

## Supplementary Material

Supplemental Figure S1 Supplemental Figure S1: Matched samples from Bone Marrow (green) and Peripheral Blood (red), testing the null hypothesis that the two specimen types come from independent random samples from normal distributions with equal means and equal but unknown variances (two-sample t-test) in our dataset. The null hypothesis is not rejected for all 16 parameters, and marked as not significantly different (N.S) for each.

Supplemental Figure S2 Supplemental Figure S2: Violin plots of Markov cell state transition rate parameters for (A) BCR::ABL1 positive patient samples, (B) BCR::ABL1 negative patient samples, and (C) molecular remission patient samples.

Supplemental Figure S3 Supplemental Figure S3. Box plots summarizing inter-patient heterogeneity in Markov transition rates. Each box plot displays the distribution of transition parameters (Mij) across patients within the specified clinical group (Diagnosis (PB), Diagnosis (BM), remission, relapse).

Supplemental Figure S4 Supplemental Figure S4. Patient-specific CTMC transition rates by disease status. Box plots compare relapse (blue) vs remission (magenta) for six estimated rates: A Other → CD34^+^/CD38^−^ (inflow to stem-like), B CD34^+^/CD38^−^ → Other (exit from stem-like), C Other → CD34^−^/CD38^+^ (inflow to differentiated), D CD34^−^/CD38^+^ → Other (exit from differentiated), E CD34^+^/CD38^−^ → CD34^−^/CD38^+^ (forward differentiation), and F CD34^−^/CD38^+^ → CD34^+^/CD38^−^ (back-conversion/dedifferentiation). Remission shows higher in E and lower in F, consistent with therapy favoring forward differentiation and limiting dedifferentiation; relapse exhibits the opposite tendency and greater variance, indicating heterogeneous mechanisms that can preserve or re-establish stemness.

Supplemental Table T1 Supplemental Table T1: Table shows the number of remission patient samples that have the corresponding Markov transition parameter statistically significantly equivalent to zero (one-sided t-test).

Supplemental Table T2 Supplemental Table T2: Table shows the number of bone marrow patient samples that have the corresponding Markov transition parameter statistically significantly equivalent to zero (one-sided t-test).

Supplemental Table T3 Supplemental Table T3: Table shows the number of peripheral blood patient samples that have the corresponding Markov transition parameter statistically significantly equivalent to zero (one-sided t-test).

Supplemental Table T4 Supplemental Table T4: Table shows the mean, median, standard deviation (sd), interquartile range (IQR) and corresponding values for first quartile (q1) and third quartile (q3) for Markov chain model trained on remission samples.

Supplemental Table T5 Supplemental Table T5: Table shows the mean, median, standard deviation (sd), interquartile range (IQR) and corresponding values for first quartile (q1) and third quartile (q3) for Markov chain model trained on relapse samples.

Supplemental Table T6 Supplemental Table T6: Table shows the mean, median, standard deviation (sd), interquartile range (IQR) and corresponding values for first quartile (q1) and third quartile (q3) for Markov chain model trained on diagnosis samples: bone marrow (BM) and peripheral blood (PB).

## Data Availability

All code and anonymized data associated with this article are publicly available at https://github.com/MathOnco/Cell-State-Transitions-B-ALL.

## References

[1] Lapidot T , Sirard C, Vormoor J, Murdoch B, Hoang T, Caceres-Cortes J, . A cell initiating human acute myeloid leukaemia after transplantation into SCID mice. Nature 1994;367:645–8. 7509044 10.1038/367645a0

[2] Bonnet D , Dick JE. Human acute myeloid leukemia is organized as a hierarchy that originates from a primitive hematopoietic cell. Nat Med 1997;3:730–7. 9212098 10.1038/nm0797-730

[3] de Grouw EPLM , Raaijmakers MHGP, Boezeman JB, van der Reijden BA, van de Locht LTF, de Witte TJM, . Preferential expression of a high number of ATP binding cassette transporters in both normal and leukemic CD34^+^CD38^−^ cells. Leukemia 2006;20:750–4. 16467867 10.1038/sj.leu.2404131

[4] Stevens BM , Jones CL, Pollyea DA, Culp-Hill R, D’Alessandro A, Winters A, . Fatty acid metabolism underlies venetoclax resistance in acute myeloid leukemia stem cells. Nat Cancer 2020;1:1176–87. 33884374 10.1038/s43018-020-00126-z PMC8054994

[5] Terwijn M , Zeijlemaker W, Kelder A, Rutten AP, Snel AN, Scholten WJ, . Leukemic stem cell frequency: a strong biomarker for clinical outcome in acute myeloid leukemia. PLoS One 2014;9:e107587. 25244440 10.1371/journal.pone.0107587 PMC4171508

[6] Hanekamp D , Denys B, Kaspers GJL, Te Marvelde JG, Schuurhuis GJ, De Haas V, . Leukaemic stem cell load at diagnosis predicts the development of relapse in young acute myeloid leukaemia patients. Br J Haematol 2018;183:512–16. 29076143 10.1111/bjh.14991

[7] van Rhenen A , Feller N, Kelder A, Westra AH, Rombouts E, Zweegman S, . High stem cell frequency in acute myeloid leukemia at diagnosis predicts high minimal residual disease and poor survival. Clin Cancer Res 2005;11:6520–7. 16166428 10.1158/1078-0432.CCR-05-0468

[8] Ho TC , LaMere M, Stevens BM, Ashton JM, Myers JR, O’Dwyer KM, . Evolution of acute myelogenous leukemia stem cell properties after treatment and progression. Blood 2016;128:1671–8. 27421961 10.1182/blood-2016-02-695312 PMC5043124

[9] Kong Y , Yoshida S, Saito Y, Doi T, Nagatoshi Y, Fukata M, . CD34^+^CD38^+^CD19^+^ as well as CD34^+^CD38^−^CD19^+^ cells are leukemia-initiating cells with self-renewal capacity in human B-precursor ALL. Leukemia 2008;22:1207–13. 18418410 10.1038/leu.2008.83

[10] Jiang Z , Deng M, Wei X, Ye W, Xiao Y, Lin S, . Heterogeneity of CD34 and CD38 expression in acute B lymphoblastic leukemia cells is reversible and not hierarchically organized. J Hematol Oncol 2016;9:94. 27660152 10.1186/s13045-016-0310-1 PMC5034590

[11] Aoki Y , Watanabe T, Saito Y, Kuroki Y, Hijikata A, Takagi M, . Identification of CD34^+^ and CD34^−^ leukemia-initiating cells in MLL-rearranged human acute lymphoblastic leukemia. Blood 2015;125:967–80. 25538041 10.1182/blood-2014-03-563304 PMC4319237

[12] Lazarus HM , Andersen J, Chen MG, Variakojis D, Mansour EG, Oette D, . Recombinant granulocyte-macrophage colony-stimulating factor after autologous bone marrow transplantation for relapsed non-Hodgkin’s lymphoma: blood and bone marrow progenitor growth studies. A phase II Eastern Cooperative Oncology Group Trial. Blood 1991;78:830–7. 1859894

[13] Lang F , Wojcik B, Bothur S, Knecht C, Falkenburg JHF, Schroeder T, . Plastic CD34 and CD38 expression in adult B–cell precursor acute lymphoblastic leukemia explains ambiguity of leukemia-initiating stem cell populations. Leukemia 2017;31:731–4. 27956738 10.1038/leu.2016.315 PMC5339428

[14] Long J , Liu S, Li K, Zhou X, Zhang P, Zou L. High proportion of CD34^+^/CD38^−^cells is positively correlated with poor prognosis in newly diagnosed childhood acute lymphoblastic leukemia. Leuk Lymphoma 2014;55:611–17. 23706103 10.3109/10428194.2013.807924

[15] Ebinger M , Witte K-E, Ahlers J, Schäfer I, André M, Kerst G, . High frequency of immature cells at diagnosis predicts high minimal residual disease level in childhood acute lymphoblastic leukemia. Leuk Res 2010;34:1139–42. 20378168 10.1016/j.leukres.2010.03.023

[16] Shman TV , Movchan LV, Aleinikova OV. Frequencies of immature CD34^+^CD38^−^ and CD34^+^CD38^−^CD19^+^ blasts correlate with minimal residual disease level in pediatric B-cell precursor acute lymphoblastic leukemia. Leuk Lymphoma 2013;54:2560–2. 23432723 10.3109/10428194.2013.778404

[17] Tabernero MD , Bortoluci AM, Alaejos I, López-Berges MC, Rasillo A, García-Sanz R, . Adult precursor B-ALL with BCR/ABL gene rearrangements displays a unique immunophenotype based on the pattern of CD10, CD34, CD13 and CD38 expresssion. Leukemia 2001;15:406–14. 11237064 10.1038/sj.leu.2402060

[18] Watcham S , Kucinski I, Gottgens B. New insights into hematopoietic differentiation landscapes from single-cell RNA sequencing. Blood 2019;133:1415–26. 30728144 10.1182/blood-2018-08-835355 PMC6440294

[19] Sha Y , Wang S, Zhou P, Nie Q. Inference and multiscale model of epithelial-to-mesenchymal transition via single-cell transcriptomic data. Nucleic Acids Res 2020;48:9505–20. 32870263 10.1093/nar/gkaa725 PMC7515733

[20] Buder T , Deutsch A, Seifert M, Voss-Böhme A. CellTrans: an R package to quantify stochastic cell state transitions. Bioinform Biol Insights 2017;11:1177932217712241. 28659714 10.1177/1177932217712241 PMC5478290

[21] Gupta PB , Fillmore CM, Jiang G, Shapira SD, Tao K, Kuperwasser C, . Stochastic state transitions give rise to phenotypic equilibrium in populations of cancer cells. Cell 2011;146:633–44. 21854987 10.1016/j.cell.2011.07.026

[22] Strobl M , Gallaher J, Robertson-Tessi M, West J, Anderson ARA. Treatment of evolving cancers will require dynamic decision support. Ann Oncol 2023;34:867–84. 37777307 10.1016/j.annonc.2023.08.008 PMC10688269

[23] West J , Robertson-Tessi M, Anderson ARA. Agent-based methods facilitate integrative science in cancer. Trends Cell Biol 2023;33:300–11. 36404257 10.1016/j.tcb.2022.10.006 PMC10918696

[24] Bull JA , Byrne HM. The hallmarks of mathematical oncology. Proc IEEE 2022;110:523–40.

[25] Angelini E , Wang Y, Zhou JX, Qian H, Huang S. A model for the intrinsic limit of cancer therapy: duality of treatment-induced cell death and treatment-induced stemness. PLoS Comput Biol 2022;18:e1010319. 35877695 10.1371/journal.pcbi.1010319 PMC9352192

[26] Foo J , Basanta D, Rockne RC, Strelez C, Shah C, Ghaffarian K, . Roadmap on plasticity and epigenetics in cancer. Phys Biol 2022;19:031501. 10.1088/1478-3975/ac4ee2 PMC9190291 35078159

[27] Gunnarsson EB , De S, Leder K, Foo J. Understanding the role of phenotypic switching in cancer drug resistance. J Theor Biol 2020;490:110162. 31953135 10.1016/j.jtbi.2020.110162 PMC7785289

[28] Dénes A , Marzban S, Röst G. Global analysis of a cancer model with drug resistance due to Lamarckian induction and microvesicle transfer. J Theor Biol 2021;527:110812. 34129816 10.1016/j.jtbi.2021.110812

[29] Zhou JX , Pisco AO, Qian H, Huang S. Nonequilibrium population dynamics of phenotype conversion of cancer cells. PLoS OnE 2014;9:e110714. 25438251 10.1371/journal.pone.0110714 PMC4249833

[30] Pedersen RK , Andersen M, Stiehl T, Ottesen JT. Understanding hematopoietic stem cell dynamics—insights from mathematical modelling. Curr Stem Cell Rep 2023;9:9–16.

[31] Watson CJ , Papula AL, Poon GYP, Wong WH, Young AL, Druley TE, . The evolutionary dynamics and fitness landscape of clonal hematopoiesis. Science 2020;367:1449–54. 32217721 10.1126/science.aay9333

[32] Moeller ME , Mon Père NV, Werner B, Huang W. Measures of genetic diversification in somatic tissues at bulk and single-cell resolution. Elife 2024;12:RP89780. 38265286 10.7554/eLife.89780 PMC10945735

[33] Pedersen RK , Andersen M, Stiehl T, Ottesen JT. Mathematical modelling of the hematopoietic stem cell-niche system: clonal dominance based on stem cell fitness. J Theor Biol 2021;518:110620. 33587928 10.1016/j.jtbi.2021.110620

[34] Boklund TI , Snyder J, Gudmand-Hoeyer J, Larsen MK, Knudsen TA, Eickhardt-Dalbøge CS, . Mathematical modelling of stem and progenitor cell dynamics during ruxolitinib treatment of patients with myeloproliferative neoplasms. Front Immunol 2024;15:1384509. 38846951 10.3389/fimmu.2024.1384509 PMC11154009

[35] Kreger J , Mooney JA, Shibata D, MacLean AL. Developmental hematopoietic stem cell variation explains clonal hematopoiesis later in life. Nat Commun 2024;15:10268. 39592593 10.1038/s41467-024-54711-2 PMC11599844

[36] Gillis N , Padron E, Wang T, Chen K, DeVos JD, Spellman SR, . Pilot study of donor-engrafted clonal hematopoiesis evolution and clinical outcomes in allogeneic hematopoietic cell transplantation recipients using a national registry. Transplant Cell Ther 2023;29:640.e1–8. 10.1016/j.jtct.2023.07.021 PMC10592088 37517612

[37] Jain P , Duddu AS, Jolly MK. Stochastic population dynamics of cancer stemness and adaptive response to therapies. Essays Biochem 2022;66:387–98. 36073715 10.1042/EBC20220038

[38] Bukkuri A . Modeling stress-induced responses: plasticity in continuous state space and gradual clonal evolution. Theor Biosci 2024;143:63–77. 10.1007/s12064-023-00410-3 38289469

[39] Vipparthi K , Hari K, Chakraborty P, Ghosh S, Patel AK, Ghosh A, . Emergence of hybrid states of stem-like cancer cells correlates with poor prognosis in oral cancer. iScience 2022;25:104317. 35602941 10.1016/j.isci.2022.104317 PMC9114525

[40] Su Y , Wei W, Robert L, Xue M, Tsoi J, Garcia-Diaz A, . Single-cell analysis resolves the cell state transition and signaling dynamics associated with melanoma drug-induced resistance. Proc Natl Acad Sci 2017;114:13679–84. 29229836 10.1073/pnas.1712064115 PMC5748184

[41] Mohammadi F , Visagan S, Gross SM, Karginov L, Lagarde JC, Heiser LM, . A lineage tree-based hidden Markov model quantifies cellular heterogeneity and plasticity. Commun Biol 2022;5:1258. 36396800 10.1038/s42003-022-04208-9 PMC9671968

[42] Burkhardt DB , San Juan BP, Lock JG, Krishnaswamy S, Chaffer CL. Mapping phenotypic plasticity upon the cancer cell state landscape using manifold learning. Cancer Discov 2022;12:1847–59. 35736000 10.1158/2159-8290.CD-21-0282 PMC9353259

[43] Cho H , Ayers K, DePills L, Kuo Y-H, Park J, Radunskaya A, . Modelling acute myeloid leukaemia in a continuum of differentiation states. Lett Biomath 2018;5(Suppl 1):S69–98. 30271874 10.1080/23737867.2018.1472532 PMC6157289

[44] Ooi QX , Plan E, Bergstrand M. A tutorial on pharmacometric Markov models. CPT Pharmacometrics Syst Pharmacol 2025;14:197–216. 39670923 10.1002/psp4.13278 PMC11812945

[45] Rockne RC , Branciamore S, Qi J, Frankhouser DE, O’Meally D, Hua W-K, . State-transition analysis of time-sequential gene expression identifies critical points that predict development of acute myeloid leukemia. Cancer Res 2020;80:3157–69. 32414754 10.1158/0008-5472.CAN-20-0354 PMC7416495

[46] Frankhouser DE , Rockne RC, Uechi L, Zhao D, Branciamore S, O’Meally D, . State-transition modeling of blood transcriptome predicts disease evolution and treatment response in chronic myeloid leukemia. Leukemia 2024;38:769–80. 38307941 10.1038/s41375-024-02142-9 PMC10997512

[47] Newton PK , Mason J, Bethel K, Bazhenova LA, Nieva J, Kuhn P. A stochastic Markov chain model to describe lung cancer growth and metastasis. PLoS One 2012;7:e34637. 22558094 10.1371/journal.pone.0034637 PMC3338733

[48] Newton PK , Mason J, Bethel K, Bazhenova L, Nieva J, Norton L, . Spreaders and sponges define metastasis in lung cancer: a Markov chain Monte Carlo mathematical model. Cancer Res 2013;73:2760–9. 23447576 10.1158/0008-5472.CAN-12-4488 PMC3644026

[49] Martínez-Rubio Á , Chulián S, Niño-López A, Picón-González R, Rodríguez Gutiérrez JF, Gálvez de la Villa E, . Computational flow cytometry immunophenotyping at diagnosis is unable to predict relapse in childhood B-cell Acute Lymphoblastic Leukemia. Comput Biol Med 2025;188:109831. 39983362 10.1016/j.compbiomed.2025.109831

[50] Stolpa W , Mizia-Malarz A, Zapała M, Zwiernik B. Can CD34^+^CD38^−^ lymphoblasts, as likely leukemia stem cells, be a prognostic factor in B-cell precursor acute lymphoblastic leukemia in children? Front Pediatr 2023;11:1213009. 37675394 10.3389/fped.2023.1213009 PMC10478575

