# Supplementary material for: Cell State Transitions Drive the Evolution of Disease Progression in B-Lymphoblastic Leukemia
Source: Cancer Res Commun. 2026 Jan 7;6(1):47–59. doi: 10.1158/2767-9764.CRC-25-0277 (PMC12775648; doi:10.1158/2767-9764.CRC-25-0277)
Supplement: Supplemental Figure S1 — Matched samples from Bone Marrow (green) and Peripheral Blood (red), testing the null hypothesis that the two specimen types come from independent random samples from normal distributions with equal means and equal but unknown variances (two-sample t-test) in our dataset. The null hypothesis is not rejected for all 16 parameters, and marked as not significantly different (N.S) for each. [file crc-25-0277_supplemental_figure_s1_suppsf1.pdf]

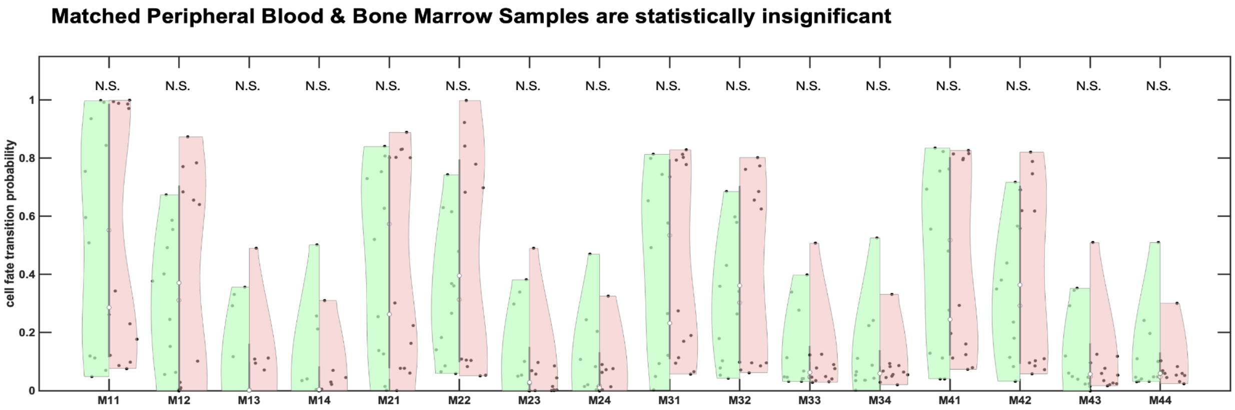

Supplemental Figure S1: Matched samples from Bone Marrow (green) and Peripheral Blood (red), testing the null hypothesis that the two specimen types come from independent random samples from normal distributions with equal means and equal but unknown variances (two-sample t-test) in our dataset. The null hypothesis is not rejected for all 16 parameters, and marked as not significantly different (N.S) for each.
