# Supplementary material for: Cell State Transitions Drive the Evolution of Disease Progression in B-Lymphoblastic Leukemia
Source: Cancer Res Commun. 2026 Jan 7;6(1):47–59. doi: 10.1158/2767-9764.CRC-25-0277 (PMC12775648; doi:10.1158/2767-9764.CRC-25-0277)
Supplement: Supplemental Figure S2 — Violin plots of Markov cell state transition rate parameters for (A) BCR::ABL1 positive patient samples, (B) BCR::ABL1 negative patient samples, and (C) molecular remission patient samples. [file crc-25-0277_supplemental_figure_s2_suppfs2.pdf]

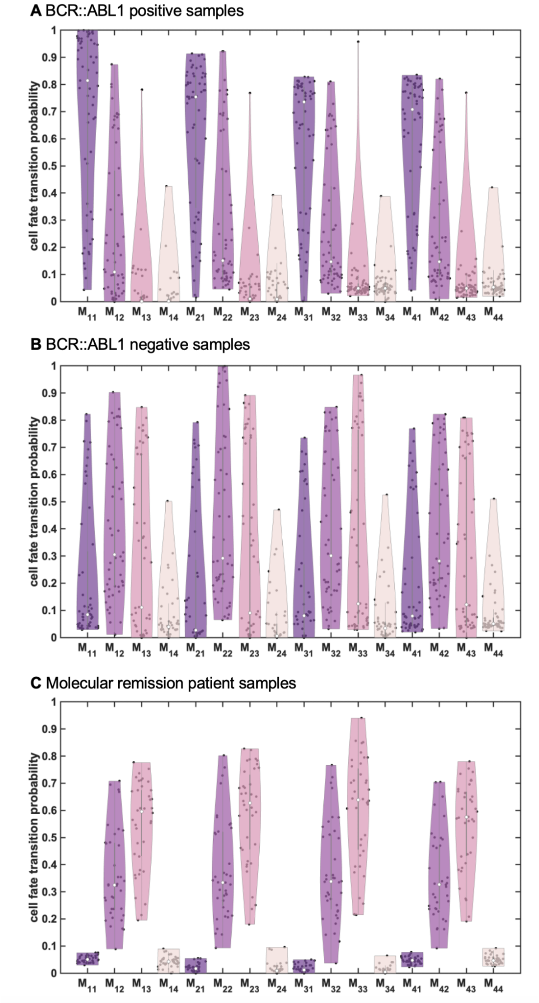

Supplemental Figure S2: Violin plots of Markov cell state transition rate parameters for (A) *BCR::ABL1* positive patient samples, (B) *BCR::ABL1* negative patient samples, and (C) molecular remission patient samples.
