# Supplementary material for: Cell State Transitions Drive the Evolution of Disease Progression in B-Lymphoblastic Leukemia
Source: Cancer Res Commun. 2026 Jan 7;6(1):47–59. doi: 10.1158/2767-9764.CRC-25-0277 (PMC12775648; doi:10.1158/2767-9764.CRC-25-0277)
Supplement: Supplemental Figure S3 — Box plots summarizing inter-patient heterogeneity in Markov transition rates. Each box plot displays the distribution of transition parameters (Mij) across patients within the specified clinical group (Diagnosis (PB), Diagnosis (BM), remission, relapse). [file crc-25-0277_supplemental_figure_s3_suppsf3.pdf]

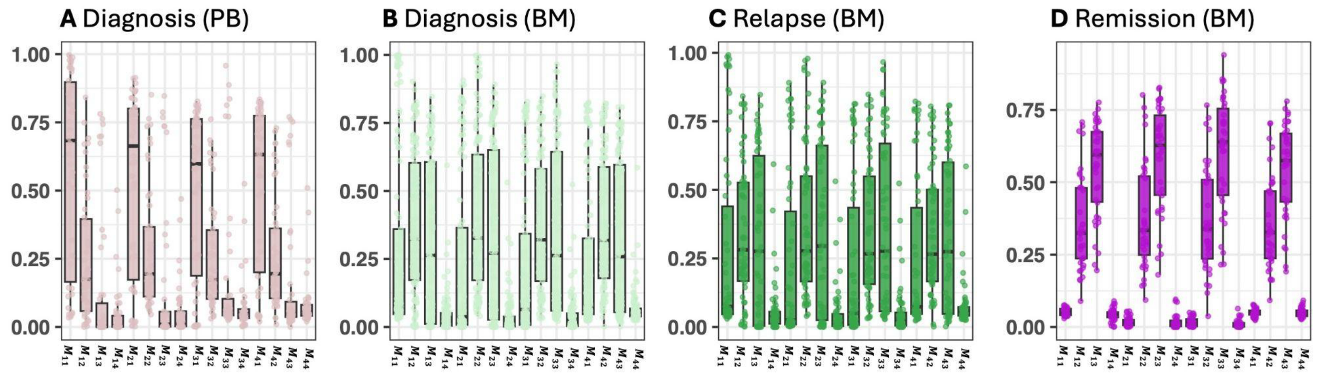

Supplemental Figure S3. Box plots summarizing inter-patient heterogeneity in Markov transition rates. Each box plot displays the distribution of transition parameters (M_{ij}) across patients within the specified clinical group (Diagnosis (PB), Diagnosis (BM), remission, relapse).
