# Supplementary material for: Cell State Transitions Drive the Evolution of Disease Progression in B-Lymphoblastic Leukemia
Source: Cancer Res Commun. 2026 Jan 7;6(1):47–59. doi: 10.1158/2767-9764.CRC-25-0277 (PMC12775648; doi:10.1158/2767-9764.CRC-25-0277)
Supplement: Supplemental Figure S4 — Patient-specific CTMC transition rates by disease status. Box plots compare relapse (blue) vs remission (magenta) for six estimated rates: A Other → CD34+/CD38− (inflow to stem-like), B CD34+/CD38− → Other (exit from stem-like), C Other → CD34−/CD38+ (inflow to differentiated), D CD34−/CD38+ → Other (exit from differentiated), E CD34+/CD38− → CD34−/CD38+ (forward differentiation), and F CD34−/CD38+ → CD34+/CD38− (back-conversion/dedifferentiation). Remission shows higher in E and lower in F, consistent with therapy favoring forward differentiation and limiting dedifferentiation; relapse exhibits the opposite tendency and greater variance, indicating heterogeneous mechanisms that can preserve or re-establish stemness. [file crc-25-0277_supplemental_figure_s4_suppsf4.pdf]

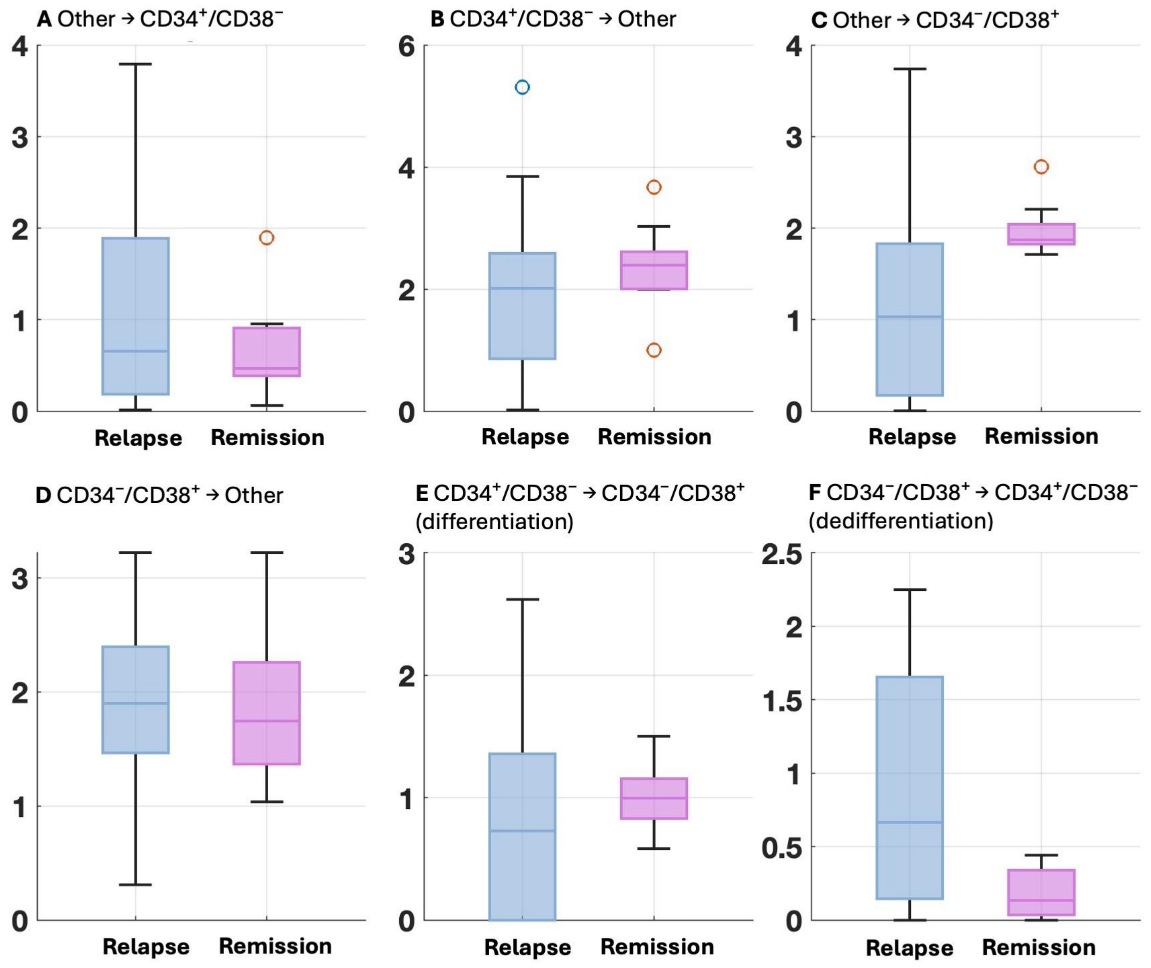

Supplemental Figure S4. Patient-specific CTMC transition rates by disease status. Box plots compare relapse (blue) vs remission (magenta) for six estimated rates: **A** *Other* → *CD34⁺/CD38⁻* (inflow to stem-like), **B** *CD34⁺/CD38⁻* → *Other* (exit from stem-like), **C** *Other* → *CD34⁻/CD38⁺* (inflow to differentiated), **D** *CD34⁻/CD38⁺* → *Other* (exit from differentiated), **E** *CD34⁺/CD38⁻* → *CD34⁻/CD38⁺* (forward differentiation), and **F** *CD34⁻/CD38⁺* → *CD34⁺/CD38⁻* (back-conversion/dedifferentiation). Remission shows higher in **E** and lower in **F**, consistent with therapy favoring forward differentiation and limiting dedifferentiation; relapse exhibits the opposite tendency and greater variance, indicating heterogeneous mechanisms that can preserve or re-establish stemness.
