# Supplementary material for: Cell State Transitions Drive the Evolution of Disease Progression in B-Lymphoblastic Leukemia
Source: Cancer Res Commun. 2026 Jan 7;6(1):47–59. doi: 10.1158/2767-9764.CRC-25-0277 (PMC12775648; doi:10.1158/2767-9764.CRC-25-0277)
Supplement: Supplemental Table T2 — Table shows the number of bone marrow patient samples that have the corresponding Markov transition parameter statistically significantly equivalent to zero (one-sided t-test). [file crc-25-0277_supplemental_table_t2_suppst2.pdf]

Supplemental Table T2: Table shows the number of bone marrow patient samples that have the corresponding Markov transition parameter statistically significantly equivalent to zero (one-sided t-test).

Bone Marrow (N=63)	Fraction of patients with zero transition rates			
	$CD34^+/CD38^-$	$CD34^+/CD38^+$	$CD34^-/CD38^+$	$CD34^-/CD38^-$
$CD34^+/CD38^-$	0	3	10	9
$CD34^+/CD38^+$	1	0	2	7
$CD34^-/CD38^+$	2	0	0	3
$CD34^-/CD38^-$	0	0	0	0
