# Supplementary material for: Cell State Transitions Drive the Evolution of Disease Progression in B-Lymphoblastic Leukemia
Source: Cancer Res Commun. 2026 Jan 7;6(1):47–59. doi: 10.1158/2767-9764.CRC-25-0277 (PMC12775648; doi:10.1158/2767-9764.CRC-25-0277)
Supplement: Supplemental Table T4 — Table shows the mean, median, standard deviation (sd), interquartile range (IQR) and corresponding values for first quartile (q1) and third quartile (q3) for Markov chain model trained on remission samples. [file crc-25-0277_supplemental_table_t4_suppst4.pdf]

Supplemental Table T4: Table shows the mean, median, standard deviation (sd), interquartile range (IQR) and corresponding values for first quartile (q1) and third quartile (q3) for Markov chain model trained on remission samples.

Feature	n	mean	median	sd	IQR	q1	q3
M11	38	0.05	0.05	0.01	0.02	0.04	0.06
M12	38	0.36	0.32	0.16	0.24	0.24	0.48
M13	38	0.55	0.60	0.16	0.24	0.43	0.67
M14	38	0.04	0.04	0.02	0.02	0.03	0.05
M21	38	0.02	0.02	0.02	0.02	0.01	0.03
M22	38	0.38	0.33	0.18	0.27	0.25	0.52
M23	38	0.59	0.63	0.18	0.28	0.46	0.73
M24	38	0.02	0.01	0.02	0.02	0.00	0.02
M31	38	0.02	0.01	0.02	0.02	0.00	0.03
M32	38	0.37	0.34	0.18	0.27	0.24	0.51
M33	38	0.60	0.64	0.19	0.30	0.46	0.75
M34	38	0.01	0.00	0.01	0.01	0.00	0.01
M41	38	0.05	0.05	0.01	0.01	0.04	0.06
M42	38	0.35	0.33	0.16	0.23	0.24	0.47
M43	38	0.55	0.57	0.16	0.24	0.43	0.67
M44	38	0.05	0.05	0.02	0.02	0.04	0.06
