# Supplementary material for: Cell State Transitions Drive the Evolution of Disease Progression in B-Lymphoblastic Leukemia
Source: Cancer Res Commun. 2026 Jan 7;6(1):47–59. doi: 10.1158/2767-9764.CRC-25-0277 (PMC12775648; doi:10.1158/2767-9764.CRC-25-0277)
Supplement: Supplemental Table T5 — Table shows the mean, median, standard deviation (sd), interquartile range (IQR) and corresponding values for first quartile (q1) and third quartile (q3) for Markov chain model trained on relapse samples. [file crc-25-0277_supplemental_table_t5_suppst5.pdf]

Supplemental Table T5: Table shows the mean, median, standard deviation (sd), interquartile range (IQR) and corresponding values for first quartile (q1) and third quartile (q3) for Markov chain model trained on relapse samples.

Feature	n	mean	median	sd	IQR	q1	q3
M11	59	0.27	0.07	0.34	0.39	0.05	0.44
M12	59	0.35	0.28	0.25	0.36	0.17	0.53
M13	59	0.32	0.28	0.29	0.62	0.00	0.62
M14	59	0.06	0.04	0.10	0.04	0.01	0.06
M21	59	0.23	0.03	0.31	0.41	0.01	0.42
M22	59	0.38	0.28	0.28	0.38	0.17	0.55
M23	59	0.34	0.30	0.31	0.64	0.03	0.66
M24	59	0.05	0.01	0.10	0.04	0.00	0.05
M31	59	0.22	0.05	0.29	0.43	0.01	0.44
M32	59	0.36	0.27	0.25	0.39	0.16	0.55
M33	59	0.37	0.28	0.31	0.61	0.06	0.67
M34	59	0.05	0.03	0.09	0.05	0.00	0.05
M41	59	0.24	0.07	0.28	0.39	0.05	0.43
M42	59	0.35	0.27	0.23	0.33	0.17	0.50
M43	59	0.33	0.27	0.28	0.55	0.05	0.60
M44	59	0.08	0.05	0.09	0.03	0.04	0.07
