# Supplementary material for: Cell State Transitions Drive the Evolution of Disease Progression in B-Lymphoblastic Leukemia
Source: Cancer Res Commun. 2026 Jan 7;6(1):47–59. doi: 10.1158/2767-9764.CRC-25-0277 (PMC12775648; doi:10.1158/2767-9764.CRC-25-0277)
Supplement: Supplemental Table T6 — Table shows the mean, median, standard deviation (sd), interquartile range (IQR) and corresponding values for first quartile (q1) and third quartile (q3) for Markov chain model trained on diagnosis samples: bone marrow (BM) and peripheral blood (PB). [file crc-25-0277_supplemental_table_t6_suppst6.pdf]

Supplemental Table T6: Table shows the mean, median, standard deviation (sd), interquartile range (IQR) and corresponding values for first quartile (q1) and third quartile (q3) for Markov chain model trained on diagnosis samples: bone marrow (BM) and peripheral blood (PB).

Specimen	Feature	n	mean	median	sd	IQR	q1	q3
BM	M11	109	0.27	0.08	0.33	0.31	0.05	0.36
PB	M11	46	0.57	0.68	0.36	0.73	0.16	0.90
BM	M12	109	0.37	0.32	0.26	0.43	0.17	0.60
PB	M12	46	0.25	0.17	0.24	0.34	0.06	0.40
BM	M13	109	0.31	0.26	0.29	0.60	0.01	0.61
PB	M13	46	0.13	0.00	0.25	0.09	0.00	0.09
BM	M14	109	0.05	0.03	0.07	0.05	0.00	0.05
PB	M14	46	0.05	0.00	0.10	0.04	0.00	0.04
BM	M21	109	0.22	0.04	0.31	0.36	0.01	0.36
PB	M21	46	0.53	0.66	0.33	0.63	0.17	0.80
BM	M22	109	0.41	0.33	0.28	0.46	0.17	0.63
PB	M22	46	0.28	0.19	0.23	0.26	0.11	0.37
BM	M23	109	0.33	0.27	0.31	0.62	0.03	0.65
PB	M23	46	0.14	0.01	0.26	0.06	0.00	0.06
BM	M24	109	0.04	0.01	0.07	0.04	0.00	0.04
PB	M24	46	0.05	0.01	0.10	0.06	0.00	0.06
BM	M31	109	0.22	0.06	0.29	0.34	0.01	0.34
PB	M31	46	0.49	0.60	0.31	0.57	0.19	0.76
BM	M32	109	0.38	0.32	0.25	0.41	0.17	0.58
PB	M32	46	0.26	0.17	0.21	0.25	0.10	0.35
BM	M33	109	0.36	0.26	0.31	0.58	0.06	0.64
PB	M33	46	0.17	0.05	0.27	0.06	0.04	0.10
BM	M34	109	0.04	0.03	0.07	0.05	0.00	0.05
PB	M34	46	0.07	0.05	0.10	0.03	0.03	0.07
BM	M41	109	0.24	0.08	0.28	0.28	0.05	0.33
PB	M41	46	0.51	0.63	0.30	0.57	0.20	0.77
BM	M42	109	0.37	0.32	0.24	0.41	0.18	0.59
PB	M42	46	0.26	0.19	0.21	0.26	0.11	0.36
BM	M43	109	0.33	0.26	0.28	0.54	0.05	0.60
PB	M43	46	0.15	0.05	0.24	0.06	0.04	0.09
BM	M44	109	0.07	0.05	0.07	0.03	0.04	0.07
PB	M44	46	0.08	0.05	0.09	0.04	0.04	0.08
